# TC2N, a novel oncogene, accelerates tumor progression by suppressing p53 signaling pathway in lung cancer

**DOI:** 10.1038/s41418-018-0202-8

**Published:** 2018-09-25

**Authors:** Xiang-lin Hao, Fei Han, Ning Zhang, Hong-qiang Chen, Xiao Jiang, Li Yin, Wen-bin Liu, Dan-dan Wang, Jian-ping Chen, Zhi-hong Cui, Lin Ao, Jia Cao, Jin-yi Liu

**Affiliations:** Institute of Toxicology, College of Preventive Medicine, Third Military Medical University, Chongqing, 400038 China

**Keywords:** Oncogenes, Non-small-cell lung cancer, Non-small-cell lung cancer, Oncogenes, Non-small-cell lung cancer

## Abstract

The protein containing the C2 domain has been well documented for its essential roles in endocytosis, cellular metabolism and cancer. Tac2-N (TC2N) is a tandem C2 domain-containing protein, but its function, including its role in tumorigenesis, remains unknown. Here, we first identified TC2N as a novel oncogene in lung cancer. TC2N was preferentially upregulated in lung cancer tissues compared with adjacent normal lung tissues. High TC2N expression was significantly associated with poor outcome of lung cancer patients. Knockdown of TC2N markedly induces cell apoptosis and cell cycle arrest with repressing proliferation in vitro, and suppresses tumorigenicity in vivo, whereas overexpression of TC2N has the opposite effects both in vitro and in vivo. Using a combination of TCGA database and bioinformatics, we demonstrate that TC2N is involved in regulation of the p53 signaling pathway. Mechanistically, TC2N attenuates p53 signaling pathway through inhibiting Cdk5-induced phosphorylation of p53 via inducing Cdk5 degradation or disrupting the interaction between Cdk5 and p53. Moreover, the blockade of p53 attenuates the function of TC2N knockdown in the regulation of cell proliferation and apoptosis. In addition, downregulated TC2N is involved in the apoptosis of lung cancer cells induced by doxorubicin, leading to p53 pathway activation. Overall, these findings uncover a role for the p53 inactivator TC2N in regulating the proliferation and apoptosis of lung cancer cells. Our present study provides novel insights into the mechanism of tumorigenesis in lung cancer.

## Introduction

Lung cancer is the leading cause of cancer-related incidents and mortality worldwide, with an estimated 1.8 million new lung cancer cases in 2012 [1]. Despite improvements in therapeutic strategies, lung cancer still has an extremely poor prognosis, with a 5-year survival rate of ~20% [2]. The late diagnosis and lack of effective therapy are the main reasons leading to the poor prognosis of patients with lung cancer [3–5]. Thus, identification of new functional genes and biomarkers in tumor development and progression may provide valuable insights into the prevention, early detection and treatment of lung cancer patients.

Tac2-N (TC2N), located on human chromosome 14q32.12, encodes a putative C2 domain-containing protein that belongs to the carboxyl-terminal type (C-type) tandem C2 protein family. TC2N was first cloned in the mouse, and this protein contains two C-terminal C2 domains, C2A and C2B [6]. The C2 domain was originally identified as a protein structural domain of calcium-dependent protein kinase C [7–9]. Further studies indicated that the function of the C2 domain is not limited to calcium-dependent phospholipid binding, since this motif has been implicated in cellular signal transduction and protein–protein interactions [10]. Moreover, a number of proteins containing C2 domains are involved in the regulation of tumorigenesis. For instance, the DOC2B gene functions as a tumor suppressor in cervical cancer through inhibiting cell proliferation, migration and invasion [11]. Myoferlin is an understudied oncogene that increases the metastasis of triple-negative breast cancer [12]. However, few studies have investigated the role and mechanism of TC2N in cancer.

In the present study, we examined the expression of TC2N in human lung tumors and matching adjacent normal tissues using tissue microarray (TMA), and found that TC2N protein level was overexpressed in human lung cancer. We further clarified the clinicopathological and prognostic significance of TC2N in lung cancer. Specifically, through in vitro and in vivo assays, we demonstrated that TC2N acts as a novel oncogene by inhibiting apoptosis and promoting the proliferation of lung cancer cells. The mechanism clarified that TC2N represses p53 transcriptional activity through inhibiting cyclin-dependent kinase 5 (Cdk5)-induced phosphorylation of p53 via inducing Cdk5 degradation or disrupting the interaction between Cdk5 and p53 in lung cancer cells.

## Results

### Expression levels of TC2N are correlated with lung cancer progression and survival of patients

**Fig. 1:**
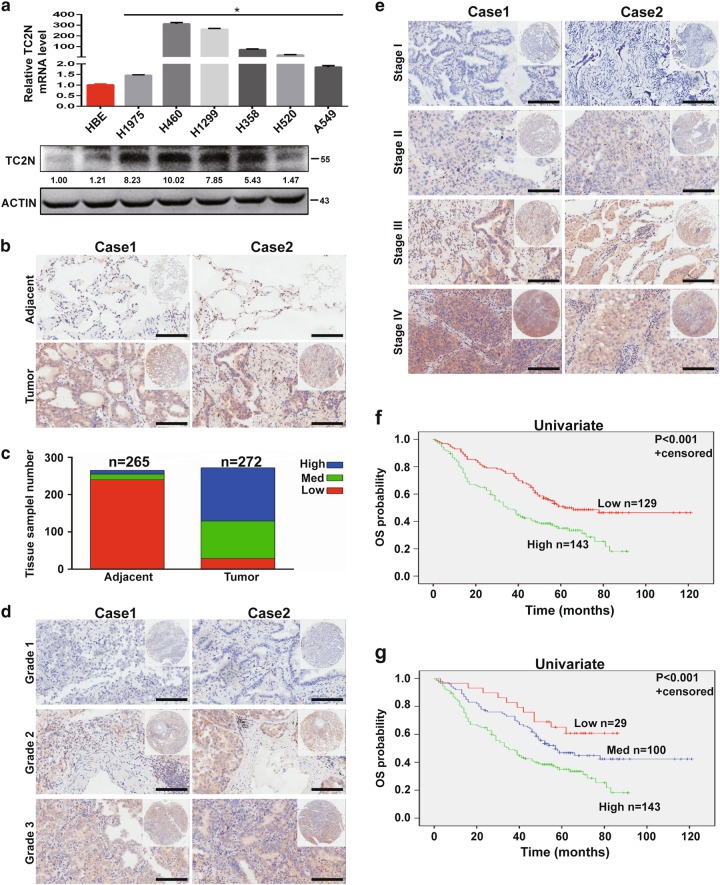
The expression level of TC2N is correlated with lung cancer progression. **a** The measurements of TC2N expression in lung cancer cell lines compared with the normal cell line HBE by qRT-PCR and WB. ACTIN is shown as a loading control. The data were analyzed using the formula 2^‒Δct^. Data represent the mean ± SD of triplicates. **b**, **c** The protein expression of TC2N in adjacent and tumor lung samples monitored by immunohistochemistry (IHC) on a tissue array. The anti-TC2N antibody was employed for IHC. IHC scoring was performed blind, prior to association with clinical data. **d** IHC analysis on a tissue array of lung cancers at different histological grades. **e** Immunohistochemistry analysis on a tissue array of lung cancers at different clinical stages. Scale bars represent 50 μm. **f** Kaplan–Meier analysis of TC2N expression in 272 lung cancer patients subdivided into two groups. **g** Kaplan–Meier analysis of TC2N expression in 272 lung cancer patients subdivided into three groups *P < 0.05

To obtain insight into the potential role of TC2N in lung cancer progression, we first monitored the messenger RNA (mRNA) and protein expression levels of TC2N in various lung cancer cell lines by quantitative PCR and western blotting (WB). As shown in Fig. 1a, TC2N was overexpressed in a variety of lung cancer cell lines compared with HBE cells (a human bronchial epithelial cell line). To further determine the expression of TC2N in lung cancer patients, we examined the levels of TC2N protein on a TMA containing 272 lung tumor tissues and 265 paired adjacent non-tumor tissues. After immunohistochemistry (IHC), we used a scoring system to consolidate the results and calculate the intensity and positive staining percentage of staining. Consistently, we also observed that TC2N was highly expressed in tumor tissues compared to peritumoral tissues (Fig. 1b, c). To further confirm these results, we subsequently analyzed TC2N mRNA expression in clinical specimens from the Selamat and Okayama lung databases (Compendia Biosciences, www.oncomine.org). The results showed that the mRNA expression of TC2N was significantly upregulated in lung tumor tissues compared with normal lung tissues (*P* < 0.001) (Supplementary Figure S1a and S1b).

After analyzing the potential associations between TC2N expression and the clinicopathological characteristics of lung cancer patients, we found that TC2N expression levels were significantly correlated with clinical stage and histological grade (Fig. 1d, e, Table 1). Further, to explore whether TC2N is a potential prognostic factor for lung cancer, we performed Kaplan–Meier survival analysis of the 272 lung cancer patients based on their TC2N expression levels. We divided all patients into two groups, the high TC2N expression group (*n* = 143) and the low TC2N expression group (*n* = 129), based on the median TC2N expression level, and the Kaplan–Meier analysis revealed a poorer clinical outcome for lung cancer patients with high TC2N expression compared to those with low TC2N expression (*P* < 0.001) (Fig. 1f). This result was further confirmed by the survival analysis of three directly obtained groups: high (*n* = 143), intermediate (*n* = 100) and low (*n* = 29) (*P* < 0.001) (Fig. 1g). Consistently, two public data sets, GSE3141 containing 111 lung cancer patients and GSE31210 containing 226 lung cancer patients, showed that high TC2N expression levels were associated with a markedly shorter overall survival (OS) of lung cancer patients (Supplementary Figure S1c, hazard ratio (HR) = 1.73, *P* = 0.046; Supplementary Figure S1d, HR = 2.79, *P* = 0.0016). Thereafter, a multivariate Cox regression analysis was performed on TMA to assess the association between TC2N and OS in the presence of clinicopathological characteristics. The multivariate analysis showed that TC2N expression was an independent prognostic factor for patient survival (HR = 1.705, *P* = 0.001), in addition to clinical stage (HR = 1.676, *P* < 0.001) and histological type (HR = 0.356, *P* < 0.001) (Table 2, Supplementary Figure S1e and S1f).

**Table 1:** Association of TC2N expression with lung cancer clinicopathological characteristics

Variable	Category	Relative TC2N expression			*P*
		High (*n* = 143)	Medium (*n* = 100)	Low (*n* = 29)	
Age (years)	<60	45	40	12	0.310
	≥60	97	59	17	
Histological type	ADC	90	73	19	0.257
	SCC	53	27	10	
**Clinical stage**	**I**	**35**	**30**	**14**	**0.006**
**(AJCC)**	**II**	**31**	**39**	**5**	
	**III**	**53**	**22**	**7**	
	IV	**1**	**1**	**1**	
Gender	Male	98	63	22	0.386
	Female	45	37	7	
**Histological grade**	**1**	**2**	**13**	**7**	<**0.001**
	**2**	**88**	**78**	**21**	
	**3**	**53**	**9**	**1**	
Tumor size	≤3cm	43	34	8	0.776
	>3 cm	89	62	20	
Lymph node status	Negative	58	44	19	0.067
	Positive	68	48	8	

Bold values indicate statistical significance, *P* < 0.05

*ADC* adenocarcinoma, *SCC* squamous cell carcinoma

**Table 2:** Multivariate analysis of different prognostic factors in human lung cancer patients (*n* = 272)

Variables	Multivariate analysis		
	HR	95% CI	P
**TC2N expression**	1.705	1.254–2.320	**0.001**
Age	1.335	0.929–1.918	0.118
Gender	1.247	0.851–1.827	0.257
**Clinical stage**	1.676	1.257–2.235	<**0.001**
**Histological type**	0.356	0.232–0.548	<**0.001**
Histological grade	1.066	0.737–1.540	0.735
Tumor size	1.356	0.903–2.035	0.142
Lymph node status	1.153	0.716–1.858	0.558

Bold values indicate statistical significance, *P* < 0.05

*HR* hazard ratio, *CI* confidence interval

### TC2N promotes lung cancer cell proliferation and inhibits apoptosis in vitro

**Fig. 2:**
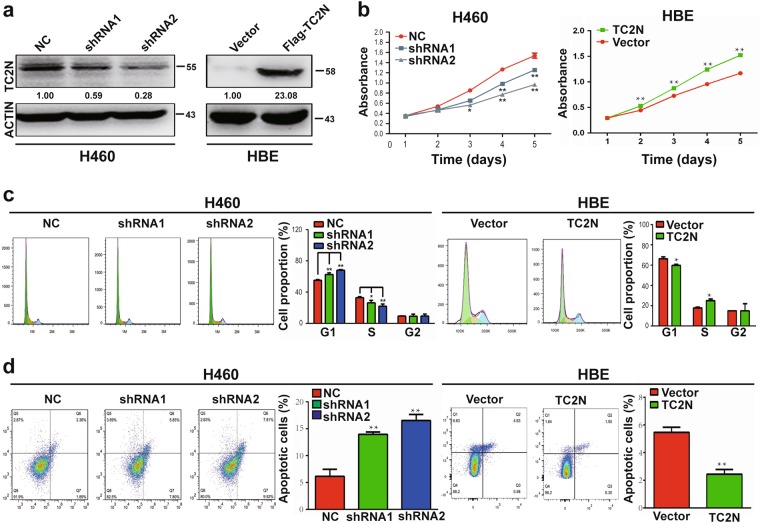
Effects of ectopic expression of TC2N on lung cancer cell proliferation and apoptosis in vitro. **a** Knockdown of TC2N in H460 cells and overexpression of TC2N in HBE cells were identified by WB assay. ACTIN serves as a loading control. **b** MTS assays were carried out in H460 cells expressing the negative control or shRNA of TC2N and in HBE cells expressing the vector control or TC2N. *P < 0.05; **P < 0.01. **c**, **d** Flow cytometry assays were used to examine the effect of TC2N on cell cycle (**c**) and cell apoptosis (**d**). Error bars indicate SD (*n* = 3)

To explore the potential role of TC2N in tumorigenesis, we transfected TC2N small hairpin RNA (shRNA) and the wild-type (WT) full-length TC2N Flag-tagged fusion vector into H460 and HBE cell lines. The expression of TC2N was verified by WB analysis (Fig. 2a). We then assessed the role of TC2N in cell proliferation and viability. The data showed that TC2N knockdown markedly impeded the proliferation of H460 cells, while TC2N overexpression promoted the growth of HBE cells (Fig. 2b, Supplementary Figure S2a), and the accelerative effect of TC2N on cell proliferation was confirmed by a colony formation assay (Supplementary Figure S2b). Consistent with this observation, the knockdown of TC2N affected cell cycle distribution and induced sub-G1 phase arrest; conversely, the overexpression of TC2N promoted cell cycle progression, which was evident by a decrease in the subpopulation of cells in sub-G1 phase (Fig. 2c). Next, to examine the effect of TC2N on cell apoptosis, Annexin V-APC/7-amino-actinomycin D double staining was performed, followed by flow cytometry analysis. The most significant findings were that the knockdown of TC2N in H460 cells significantly increased the percentage of early apoptotic cells and late apoptotic cells and that the overexpression of TC2N inhibited HBE cell apoptosis (Fig. 2d). Similar results were also obtained when TC2N was transfected into A549 and H1975 cell lines (Supplementary Figure S3). These data together with the aforementioned results suggested that TC2N might act as a potential oncogene in lung cancer.

### TC2N promotes the tumorigenic behavior of lung cancer cells in vivo

**Fig. 3:**
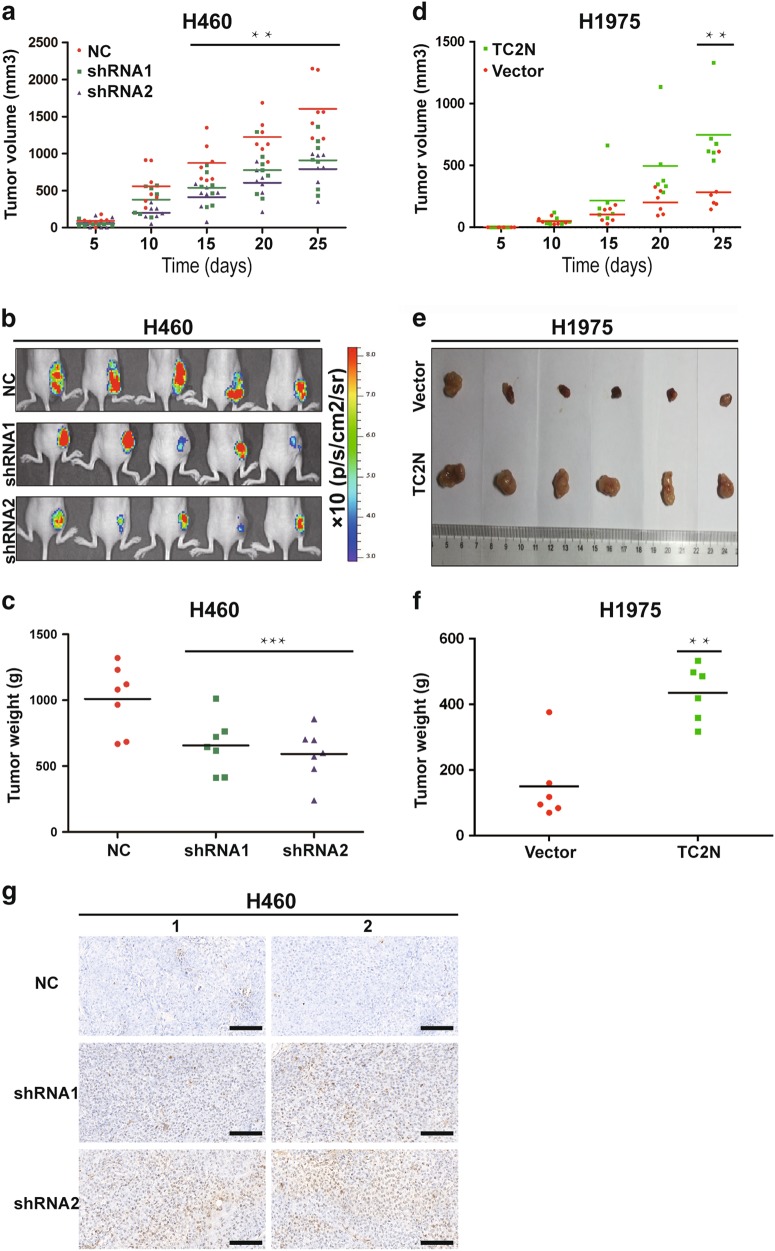
TC2N promotes tumorigenesis of lung cancer cells in nude mice. **a** Examination of tumorigenesis in subcutaneously injected animals using H460 cells stably expressing the negative control or TC2N shRNA. The tumor growth curve of shRNA-expressing cells was compared with negative control cells. **b** The fluorescent images of xenograft tumors from nude mice subcutaneously injected with H460 stable transfectants were photographed. **c** Tumor weight from the negative control or TC2N shRNA groups. Each group contained seven mice. **d** The tumor growth curve was performed in nude mice using H1975 cells stably expressing vector control or TC2N. **e** Xenograft tumors from (**d**) were dissected and photographed. **f** Tumor weight from the vector control and TC2N groups. Each group contained six mice. **g** Knockdown of TC2N increases the number of apoptotic H460 cells in xenograft tumors. Two representative images were randomly selected from each group (H460-NC, H460-shRNA1 and H460-shRNA2). Scale bars represent 50 μm *P < 0.05; **P < 0.01;***P < 0.001

Due to the fact that HBE is a non-tumorigenic normal bronchial epithelial cell line, we used H460 and H1975 cell lines to investigate the biological functions of TC2N in vivo. As shown in Supplementary Figure S4, the ablation of TC2N expression in H460 cells stably expressing the shRNA vector with the enhanced green fluorescent protein (EGFP) tag was achieved. Then, these cells were subcutaneously implanted in nude mice, and tumor growth was subsequently quantified. TC2N knockdown significantly reduced tumor growth (Fig. 3a). The fluorescence levels of EGFP, indicative of tumor size, were decreased after inoculation with shRNA H460 cells compared to negative control (NC) H460 cells (Fig. 3b). Knockdown of TC2N resulted in a decrease of the mean weight of tumors (Fig. 3c). Further, TC2N overexpression accelerated the formation of irregularly shaped tumors and increased the mean weight of tumors after the injection of H1975 cells (Fig. 3d–f). Furthermore, we used xenograft tumor tissues to detect cell apoptosis by terminal deoxynucleotidyl transferase-mediated dUTP nick end labeling (TUNEL) staining. The results revealed that the knockdown of TC2N resulted in an increase in apoptotic cells in tumor tissues (Fig. 3g). Together, these data further suggested that TC2N significantly promotes lung cancer cell tumorigenesis and growth in vivo.

### TC2N inhibits p53 signaling in lung cancer cells

**Fig. 4:**
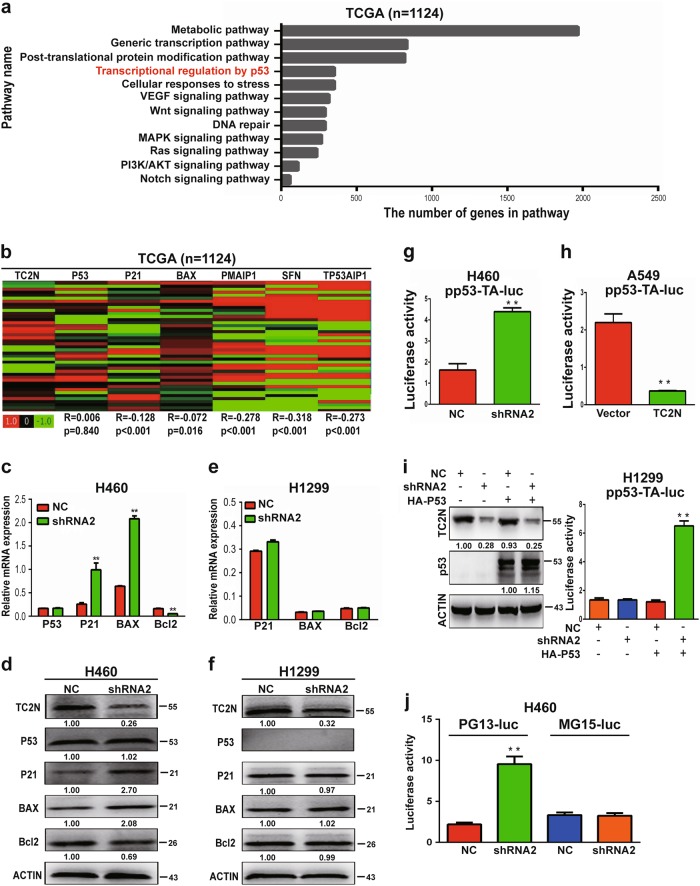
TC2N involves in regulating p53 signaling pathway. **a** Identification of potential pathways downstream of TC2N with the TCGA lung cancer RNAseq (IlluminaHiSeq; *n* = 1124) data set, as described in the Materials and methods. **b** Heatmaps for correlations between TC2N and p53 signaling pathway-related genes in the TCGA lung cancer RNAseq (IlluminaHiSeq; *n* = 1124) data set. Correlation coefficient *R* and *P* values were calculated by Spearman's correlation analysis. **c** qRT-PCR analysis of P53, P21, BAX and Bcl-2 expression in H460 cells transiently transfected with the negative control or TC2N shRNA. ACTIN serves as an internal control. **d** The protein levels of TC2N, p53, P21, BAX and Bcl-2 were monitored by WB after knockdown of TC2N in H460 cells. **e** qRT-PCR analysis of P21, BAX and Bcl-2 expression in H1299 cells transiently transfected with the negative control or TC2N shRNA. **f** The protein levels of TC2N, p53, P21, BAX and Bcl-2 were monitored by WB after knockdown of TC2N in H1299 cells. ACTIN serves as an internal control. **g** The effects of TC2N knockdown on the p53 response reporter construct pp53-TA-Luc in H460 cells. **h** The effects of TC2N overexpression on the p53 response reporter construct pp53-TA-Luc in A549 cells. **i** H1299 (p53 null) cells were co-transfected with p53 response reporter construct pp53-TA-Luc, negative control or TC2N shRNA and HA-p53 expression vectors. Luciferase activity was measured at 24 h after the transfection. **j** H460 cells were co-transfected with PG13-luc or MG15-luc reporters and TC2N expression vectors as indicated, and luciferase activity was measured 24 h after transfection. Results are means ± SEM of three independent experiments **P < 0.01

The critical role of TC2N in tumor progression motivated us to identify the genes that are regulated by TC2N. To further investigate the downstream targets involved in TC2N-mediated tumor progression, we analyzed the correlation between TC2N and other genes in lung cancer patients using The Cancer Genome Atlas (TTCGA) database. The expression levels of 13,943 genes showed significant correlations with TC2N (*P* < 0.05) (Dataset 1). Further, Reactome pathway enrichment analysis was conducted based on these 13,943 genes, revealing the top 10 enrichment-related pathways (Fig. 4a). We then analyzed the relationship between TC2N and these signaling pathways and found a significant negative correlation between TC2N and most genes of the metabolic, Wnt, vascular endothelial growth factor (VEGF), mitogen-activated protein kinase (MAPK) signaling pathway (Supplementary Table S1), which means these signaling pathway are not the targets of TC2N. As the p53 signaling pathway plays an important role in the regulation of tumor progression [13], we speculated that TC2N might promote lung tumorigenesis by repressing the p53 signaling pathway. Thus, we further analyzed the correlation between the expression of TC2N and that of p53 target genes using TCGA data. We found a significant negative correlation between TC2N and p53 signaling target genes, such as p21, BAX, PMAIP1, SFN and TP53AIP1, but there was no correlation of TC2N with p53 (Fig. 4b). As target genes p21, BAX, PMAIP1, SFN and TP53AIP1 play critical roles in cell proliferation, cell cycle and cell apoptosis, we hypothesized that TC2N may promote tumor progression through inhibition of the p53 signaling pathway. To examine this hypothesis, we measured the expression changes of p53 signaling pathway genes in shRNA-transfected H460 cells (wild-type p53) by quantitative reverse transcription-polymerase chain reaction (qRT-PCR) and WB analyses. Consistently, the knockdown of TC2N changed the expression of p53 target genes but not that of p53 (Fig. 4c, d). Furthermore, the knockdown of TC2N in H1299 cells (p53 null) did not change the expression of p53 target genes (Fig. 4e, f).

To further verify whether TC2N represses p53 transcriptional activity, we used the p53 response reporter vector pp53-TA-Luc to monitor the activation of the p53 pathway. The data showed that TC2N knockdown augmented the transcriptional activity of p53 in H460 cells, while TC2N overexpression inhibited p53-mediated transcription measured in A549 cells (Fig. 4g, h). To further confirm this result, we analyzed the transcriptional activity of p53 in p53-null H1299 cells transfected with TC2N shRNA alone or together with the pCDNA3.1-HA-p53 (HA-p53) expression vector. Consistent with the above observation, the knockdown of only TC2N expression had no effect on p53 transcriptional activity. However, the knockdown of TC2N notably increased the luciferase activity of pp53-TA-Luc after co-transfection of the HA-p53 expressing vector compared with the transfection of only p53 (Fig. 4i). Similar results were obtained with PG13-luc (wild-type p53-binding sites) and MG15-luc (mutated p53-binding sites) p53-responsive luciferase reporters. TC2N knockdown also markedly increased endogenous p53 activity from the PG13-Luc vector in H460 cells but had no effect on the activity of MG15-Luc, which cannot be regulated by p53 (Fig. 4j). Taken together, these results indicate that the TC2N-mediated inhibition of the p53 signaling pathway relies on p53 activation.

### TC2N alleviates Cdk5-induced p53 phosphorylation

**Fig. 5:**
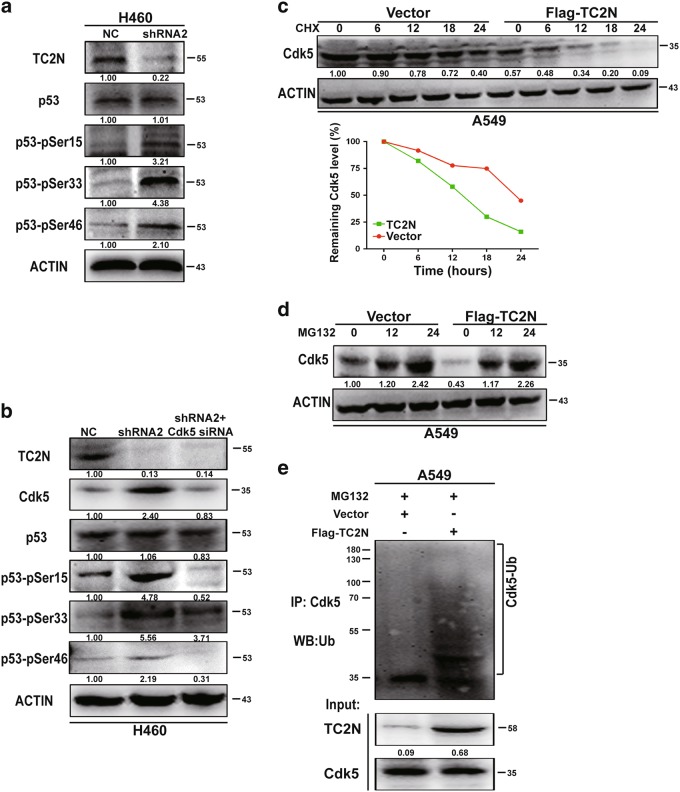
TC2N blocks Cdk5-mediated p53 phosphorylation by promoting the degradation of Cdk5. **a** H460 cells were transfected with negative control or TC2N shRNA for 48 h and then cells were lysed and were subjected to WB using anti-phospho-p53 antibodies. **b** H460 cells were co-transfected with negative control or TC2N shRNA and Cdk5 siRNA. At 48 h after transfection, the cells were lysed and were subjected to WB using indicated antibodies. **c** Lysates from A549 vector control or TC2N cells treated with 20 µM cycloheximide (CHX) for the indicated times were subjected to WB (Up). Relative Cdk5 levels were quantified by ImageJ software (Down). **d** Lysates from A549 vector control or TC2N cells were treated with 20 µM MG132 for the indicated times were subjected to WB. **e** A549 vector control or TC2N cells were transfected with 7.5 µg of ubiquitin-expressing plasmids. At 24 h after the transfection, cells were treated 20 µM MG132 for 24 h. Cell lysates were IP with anti-Cdk5 antibody and analyzed by WB with anti-ubiquitin antibody, and normal IgG was used as a negative control. Whole-cell lysates were used as a positive control (Input)

Since TC2N had an inhibitory effect on p53-mediated transcriptional activity with no significant change in the p53 protein level, we tried to figure out how this effect was achieved. Notably, p53 activity is largely controlled by post-translational modifications, such as phosphorylation [14]. Therefore, we examined whether TC2N regulates the phosphorylation of p53. Indeed, the knockdown of TC2N significantly enhanced p53 phosphorylation at Ser-15, Ser-33 and Ser-46 in H460 cells (Fig. 5a). A previous study showed that Cdk5 mediates p53 activity through inducing p53 phosphorylation at Ser-15, Ser-33 and Ser-46 in vitro [15, 16]. To further investigate whether the TC2N-dependent inhibition of p53 phosphorylation was indeed mediated by Cdk5, we knocked down Cdk5 expression using Cdk5 small interfering RNA (siRNA) with the simultaneous knockdown of TC2N in H460 cells. The knockdown of Cdk5 significantly abrogated the TC2N knockdown-induced phosphorylation of p53 at Ser-15, Ser-33 and Ser-46, suggesting that Cdk5 is involved in the TC2N-regulated dephosphorylation of p53 (Fig. 5b).

### TC2N regulates CDK5 protein stability

Interestingly, the Cdk5 protein level was markedly upregulated after TC2N knockdown, whereas the mRNA level of Cdk5 was not affected (Fig. 5b and Supplementary Figure S5). Therefore, the regulation of CDK5 by TC2N is unlikely at the transcriptional level. To test whether TC2N regulates CDK5 at the posttranscriptional level, WB assay was performed. As shown in Fig. 5c, the half-life of CDK5 protein was notably shortened when the TC2N expression was overexpressed in A549 cells. Furthermore, TC2N overexpression with MG132 treatment resulted in a remarkable accumulation of Cdk5 proteins in A549 cells (Fig. 5d), suggesting that TC2N is involved in the regulation of CDK5 stability.

The ubiquitin proteasome pathway is the common mechanism for protein degradation [17]. We next determined whether TC2N induces Cdk5 degradation by promoting its ubiquitination in lung cancer cells. Cdk5 was immunoprecipitated with specific anti-Cdk5 antibodies, and its ubiquitination status was analyzed with anti-ubiquitin antibody. As expected, the overexpression of TC2N significantly increased the ubiquitination level of Cdk5 in A549 cells (Fig. 5e).

### TC2N inhibits the interaction between Cdk5 and p53

**Fig. 6:**
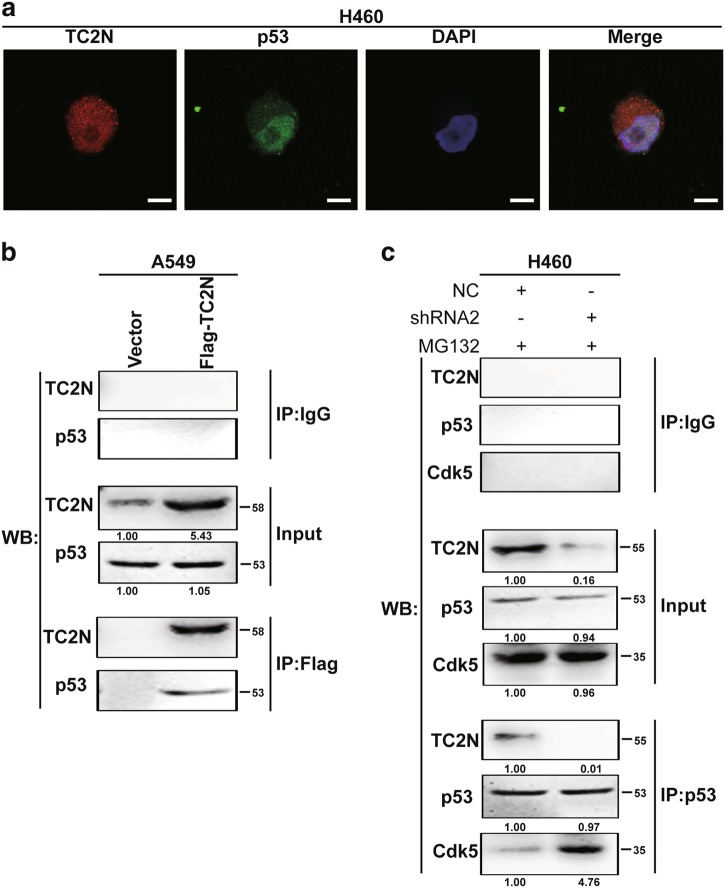
TC2N inhibits the interaction between Cdk5 and p53. **a** Immunofluorescence and microscope analysis of TC2N (red) and endogenous p53 (green) localization in nucleus of H460 cells. DAPI (blue) serves as a nuclear counterstain. Right panels show merged images. Scale bars represent 10 μm. **b** Lysates from A549 vector control or TC2N cells were IP with anti-Flag antibody, and normal IgG was used as a negative control. Whole-cell lysates were used as a positive control (Input). **c** H460 negative control or TC2N shRNA cells were treated with 20 µM MG132 for 24 h. Cell lysates were IP with anti-p53 antibody and analyzed by WB using indicated antibody

It is well established that the binding of Cdk5 to p53 induces the activation of p53 signaling pathway [16]. We then investigated whether TC2N disrupts the Cdk5–p53 interaction. Fluorescent microscopy revealed co-localization of endogenous p53 and TC2N proteins in the nucleus of H460 cells (Fig. 6a). This raised the possibility that TC2N and p53 interact or are in the same complex. To extend the above observations, we performed a co-immunoprecipitation assay. p53 can be co-immunoprecipitated with TC2N in A549 cells using anti-Flag antibody (Fig. 6b). Next, we investigated whether TC2N interferes the Cdk5–p53 interaction using anti-p53 antibody. Indeed, when TC2N was knocked down in H460 cells, the interaction between p53 and Cdk5 was detected more robustly (Fig. 6c). These results demonstrate that TC2N interferes with the binding of Cdk5 to p53.

### The function of TC2N in regulating proliferation, cell cycle and apoptosis relies on p53

**Fig. 7:**
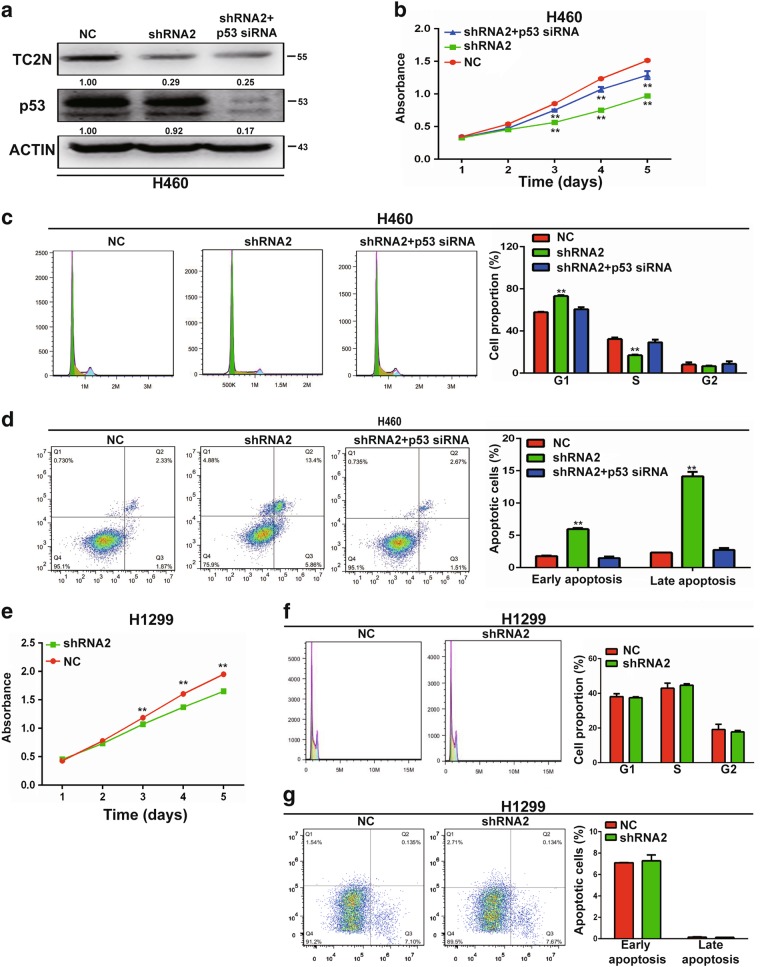
p53 is a functionally important target protein of TC2N in lung cancer cells. **a** TC2N and p53 knockdown were confirmed by WB in H460 cells. **b** MTS assays were performed to analyze cell proliferation of H460 cells co-transfected with P53 siRNA and TC2N shRNA. **P < 0.01. **c**, **d** Assessment of the effects of the expression of TC2N and P53, respectively, on cell cycle (**c**) and cell apoptosis (**d**) in H460 cells. **e** MTS assays were performed to analyze cell proliferation of H1299 cells transfected with negative control or TC2N shRNA. **f**, **g** Assessment of the effects of the overexpression of TC2N on cell cycle (**f**) and cell apoptosis (**g**) in H1299 cells

We next examined whether p53 mediates the effect of TC2N on tumorigenesis in lung cancer cells. The expression of p53 was blocked using siRNA in H460 cells with the simultaneous knockdown of TC2N (Fig. 7a), and subsequently, cell proliferation, cell cycle and apoptosis were assessed. We found that the knockdown of p53 by siRNA significantly abrogated the effects on cell proliferation, cell cycle arrest and apoptosis induced by TC2N knockdown in H460 cells (Fig. 7b–d). To further confirm this result, we knocked down the expression of TC2N in p53-null H1299 cells, whereas no change in cell cycle and apoptosis except proliferation was observed when TC2N was silenced in H1299 cells (Fig. 7e–g). These data indicate a strong functional relationship between TC2N and p53.

### TC2N knockdown increases doxorubicin-induced p53 phosphorylation and apoptosis

**Fig. 8:**
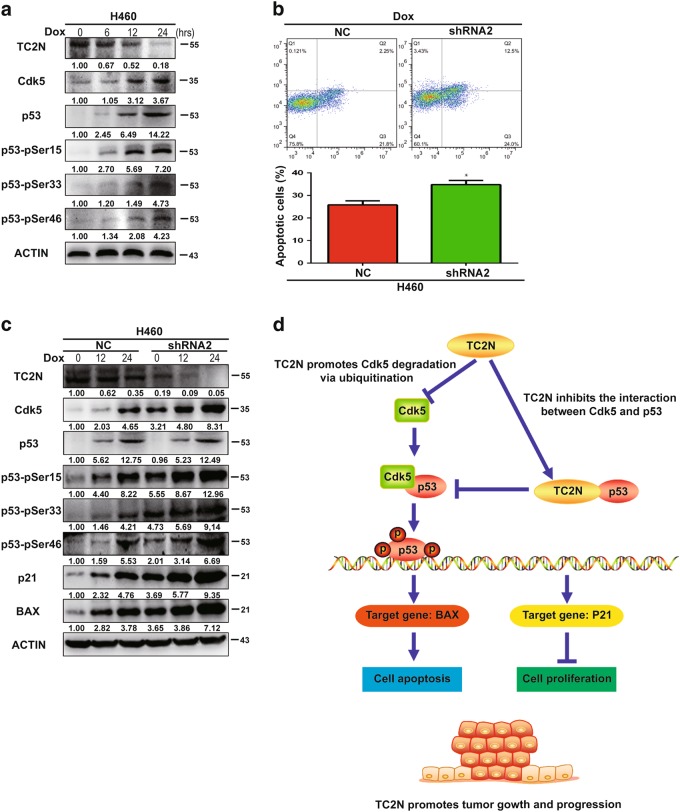
Inhibition of TC2N increases cell apoptosis with doxorubicin treatment. **a** H460 cells were incubated with 1 µM Dox for 0 to 24 h, as indicated. Cells were harvested, and then indicated protein levels were determined by WB. **b** H460 cells stably expressing the negative control or TC2N shRNA were treated with Dox (1 µM) for 24 h and then analyzed by flow cytometry for apoptosis. Results are the mean ± SEM of three independent experiments. *P < 0.05. **c** The protein expression determined by WB were depicted for the cells treated as the same in (**b**). **d** Schematic diagram for the potential mechanisms of TC2N-mediated promotion of tumorigenesis in lung cancer

Previous studies have reported that doxorubicin (Dox)-induced toxicity is mediated by p53 [18]. However, the role of TC2N in this process is unclear. We next investigated whether TC2N is involved in the Dox-induced apoptosis of lung cancer cells. The results showed that Dox treatment significantly reduced the expression of TC2N and induced the phosphorylation of p53 in H460 cells, indicating that Dox negatively regulates the TC2N/p53 axis in lung cancer cells (Fig. 8a). Using the H460 cell line, we found that knockdown of TC2N expression significantly enhanced Dox-induced apoptosis, suggesting that TC2N may play an important role in the chemoresistance of lung cancer cells (Fig. 8b). To further determine the mechanism of TC2N function in the chemoresistance of lung cancer cells, we studied the effect of TC2N knockdown on Dox-induced p53 phosphorylation. As shown in Fig. 8c, the knockdown of TC2N significantly enhanced Dox-induced p53 phosphorylation, accumulation of BAX and p21 expression in H460 cells.

## Discussion

A previous characterization of TC2N in mice revealed that the C-terminal domain of this protein contains C2A and C2B domains [6]. TC2N is structurally similar to myoferlin, a transmembrane protein containing multiple C2 domains that play roles in regulating membrane repair, tyrosine kinase receptor function, endocytosis and tumorigenesis [12, 19–24]. However, it is not clear whether TC2N has any role in cancer. In this study, we identified TC2N as a novel oncogene acting through suppression of p53 signaling pathway in human lung cancer. As compared with the HBE cell line, we found that TC2N was upregulated in most of the lung cancer cell lines, especially the H460, H358 and H1299 cell lines. Based on data from American type culture collection (ATCC), H1299 and H358 cell lines with high degree of malignancy because these cells derived from metastatic site of lung cancer patient. H460 cell belongs to large-cell neuroendocrine carcinoma of the lung, a rare subtype of non-small cell lung cancer (NSCLC), is considered to be very aggressive, and clinical outcome is poorer than expected for stage-matched NSCLC, similar to the dismal outcome of small cell lung cancer (SCLC), with 5-year survival rates ranging between 15 and 60% [25, 26]. Subsequently, we identified TC2N is significantly overexpressed in human lung cancer patients and is significantly correlated with clinical stage and histological grade. Therefore, we think that the high TC2N expression may have relationship with the degree of tumor malignancy. In addition, it is worth noting that we are performing a larger case–control study of lung cancer including more detailed and comprehensive clinicopathological data.

Subsequently, we investigated the functions of TC2N in vitro and in vivo, and the results demonstrated that the overexpression of TC2N could increase the number of cells compared with the vector control transfectants. The proliferation rate of cancer cells are the result of imbalances in proliferation on the one hand, and the induction or inhibition of apoptosis on the other [27]. Thus, we explored whether TC2N can regulate cell cycle and apoptosis of lung cancer cells. Further experiments revealed TC2N can also inhibit cell apoptosis. Taken together, TC2N caused an increase in the number of cells by enhancing the proliferation ability and inhibiting apoptosis of lung cancer cells.

The tumor suppressor p53, referred to as the “guardian of the genome and policeman of oncogenes”, plays a central role in sensing and reacting to DNA damage and oncogenic signaling [28]. Under normal conditions, p53 shows low expression because MDM2 stimulates the nuclear export of p53 and targets p53 for ubiquitination and degradation [29, 30]. Under stress, p53 is rapidly accumulated in the cell and transcriptionally regulates its target genes [31], including BAX [32], p21 [33, 34], BCL-2 [35] and PMAIP [36]. Accumulating evidence suggests that the stability and activity of p53 can be regulated through post-translational modifications, such as phosphorylation [37]. Previous results suggest that both the stability and activity of p53 are controlled by multiple protein kinases [38–41]. For instance, Cdk5 functions as an important upstream activator of p53 phosphorylation in cells [42]. Upon activation, p53 is phosphorylated by Cdk5 on Ser-15, Ser-33 and Ser-46, which enhances its transcriptional activity and target gene expression [16].

In the present study, TC2N was negatively correlated with p53 target gene expression in lung cancer tissue, and the knockdown of TC2N reduced the expression of BCL-2, but increased the expression of p21 and BAX in H460 cells. Interestingly, TC2N did not regulate the expression level of p53, but inhibited p53-medicated transcriptional activity. To obtain further insight into the mechanism of TC2N in repressing the transcriptional activity of p53, we screened for potential phospho-p53 sites that were modulated by TC2N. The knockdown of TC2N induced a higher level of p53 phosphorylation at Ser-15, Ser-33 and Ser-46. Based on earlier reports, we suspected that TC2N might impair the ability of Cdk5 to phosphorylate p53. Subsequently, we demonstrated that TC2N could reverse the Cdk5-mediated increase in p53 phosphorylation through inhibiting Cdk5 expression. However, this result showed that Cdk5 protein levels, but not mRNA levels, were increased following TC2N knockdown, indicating that the induction of Cdk5 upon TC2N knockdown occurred at the posttranscriptional level. Our further studies indicated that TC2N promotes Cdk5 degradation via ubiquitination pathway. Moreover, TC2N can also block the Cdk5-mediated p53 phosphorylation via disrupting the interaction between Cdk5 and p53.

Previous studies have shown that TP53 is the most frequently mutated in lung cancer [43, 44]. More importantly, mutant p53 not only losses transcription function, but also makes cell malignant transformation [45], motivating us to explore what role does TC2N plays in lung cancer patients with p53 mutation. Our in vitro experiments revealed that TC2N has the same biological effect on proliferation and apoptosis of H1975 cell line (with R273H mutated p53). Based on earlier studies, R273H mutated p53 activates multiple signaling pathways to induce oncogenicity in lung cancer cells by transcriptional repression of the target genes, such as P21 and BAX [46]. Thus, we investigated whether TC2N can also repress the expression of P21 and BAX in H1975 cell line. Indeed, the overexpression of TC2N decreased the expression of p21 and BAX in H1975 cells (Supplementary Figure S6). Hence, we suspect that TC2N promotes proliferation and inhibits apoptosis of H1975 cells may be via a R273H mutated p53-mediated signaling pathway. Then, it deserves to be further investigated.

Moreover, we report that TC2N is involved in doxorubicin-induced apoptosis by inhibiting p53 phosphorylation and p21 and BAX expression in lung cancer. This result suggested that TC2N may have a critical role in the chemoresistance of lung cancer. Notably, this hypothesis should be empirically tested, and further studies are required to address this aim.

Taken together, we have identified the novel functional oncogene TC2N in lung cancer. TC2N promotes the proliferation and inhibits the apoptosis of lung cancer cells, acting through the repression of p53 function in a transcription-dependent manner by inhibiting Cdk5-induced p53 phosphorylation. Moreover, TC2N may be a potential target and clinical marker for lung cancer therapy.

## Materials and methods

### Cell lines

The lung cancer cell lines (H1975, H460, H1299, H358 and H520) and HBE bronchial epithelial cell line were obtained from the Cell Bank of the Chinese Academy of Science (Shanghai, China) and the ATCC (Manassas, VA, USA), cultured in RPMI 1640 medium supplemented with 10% fetal bovine serum (Gibco, CA). All the cells were maintained at 37 °C with 5% CO_2_.

### Tissue microarray and immunohistochemical analysis

Tissue microarrays containing a total of 272 lung cancer patient tissue samples including 182 adenocarcinomas (ADCs) and 90 squamous cell carcinomas (SCCs) were obtained from the collaboration (Shanghai Biochip Co. Ltd, Shanghai, People’s Republic of China). The rabbit polyclonal antibodies used were anti-TC2N (1:500; Abcam Inc., Cambridge, MA, USA). Based on IHC, positive staining was quantified and classified into 5 categories: <10% positive cells for 0 (score); 10 to 25% for 1; 26 to 50% for 2; 51 to 75% for 3; and ≥76% for 4. Staining intensity was graded as negative (scored as 0), weak (1), moderate (2) or strong (3). All core biopsies were independently reviewed by two pathologists, and expression levels were defined by the sum of the grades for the percentage of positive staining and intensity. The clinical and pathological features of these patients are described in Supplementary Table S2.

### Plasmid construction and cell transfection

The expression vector encoding full-length open reading frame of human TC2N were constructed by synthesis and inserted into p3xFLAG-CMV-14. For knockdown, two pairs of oligomeric single-stranded oligonucleotides and a pair of negative oligomeric single-stranded oligonucleotides were synthesized, and then inserted into shRNA expression vector GV248. pp53-TA-Luc, PG13-Luc and MG15-Luc reporters were purchased from Addgene.

Cells were transfected using Lipofectamine 2000 Reagent (Invitrogen Preservation, Carlsbad, CA, USA) according to the manufacturer’s instructions. The stably transfected cells were screened under G418 (Calbiochem, La Jolla, CA, USA) or Puromycin (Sigma). Cell clones were obtained by the limited diluted method.

### RT-PCR and qRT-PCR analysis

The RNA of cells was isolated using the Trizol reagent (Invitrogen, Life Technologies), and conversion of total RNA to complementary DNA was performed with the Reverse Transcription System (Promega, Madison, WI, USA). RT-PCR was performed using the Master mixes (Promega, Madison, WI, USA) following the manufacturer’s instructions. All qRT-PCR reactions were performed using the C1000 Real-Time Cycler (Bio-Rad Laboratories, Hercules, CA, USA) and qRT-PCR Master mixes (Promega, Madison, WI, USA). Primers for amplification of the TC2N, p53, p21, BAX, Bcl-2 and ACTIN genes are listed in Supplementary Table S3. All experiments were carried out in triplicate, and the 2^‒ΔΔct^ method was used to determine expression of the genes of interest.

### MTS and EdU assay

For overexpression, HBE, A549 and H1975 cells were plated at 4 × 10^3^ cells per well on 96-well plates, and transfected with TC2N or vector control. For knockdown, H460 and H1299 cells were plated at 4 × 10^3^ cells per well on 96-well plates, and transfected with TC2N shRNA or negative control. Cell proliferation was assessed using MTS Reagent (Promega, Madison, WI, USA) on days 1, 2, 3, 4 and 5 after transfection. The assays were performed in triplicate.

The effect of TC2N on cell proliferation was also assessed by the 5-ethynyl-2′-deoxyuridine (EdU) assay. Cells were seeded into 96-well plates at a density of 4 × 10^3^ cells per well and transfected with vector for 46 h. Then, EdU assays was performed as previously described [47]. Different groups of confluent cells were randomly selected from each sample image.

### Colony formation assay

Stable transfected H1975 cells (*n* = 300) or H460 cells (*n* = 300) or A549 cells (*n* = 300) were seeded into 6-well plates and maintained in media for 14 days. Surviving colonies were fixed with 4% paraformaldehyde and stained with 0.1% crystal violet (Beyotime Biotechnology, China) for 15 min. Colonies with greater than 50 cells were counted manually. The experiment was carried out in triplicate wells for three times.

### Flow cytometry assay

Flow cytometry assay was performed as previously described [48]. Cells were seeded in 6-well plates and cultured for 24 h at 37 °C. After serum starvation for 24 h, cells were transfected with plasmid or siRNA. Then, the cells were harvested at 48 h post transfection, and analyzed by a FACSCalibur (BD Biosciences, Franklin Lakes, NJ, USA). Cell apoptosis and cell cycle profiles were determined using the ModFitLT software (Becton Dickinson, San Diego, CA, USA). All experiments were conducted three times in triplicate.

### In vivo tumorigenicity and TUNEL assay

For the xenograft tumor growth assay, a total of 1 × 10^6^ stable transfected H460 cells suspended in 150 μl PBS were injected subcutaneously into the right flanks of 5-week-old male Balb/c nude mice, respectively. Tumors size were measured every 3–5 days with calipers after injection, and the tumor volume was calculated based on formula: 0.5 × (length × width^2^). After 25 days, TUNEL assay was performed as previously described [47]. The assay was carried out for three independent experiments.

### Luciferase reporter assay

The cells (*n* = 5 × 10^4^) were grown in 24-well plates in triplicate for each condition and transfected with indicated plasmids. At 24 h after transfection, the cells were lysed to measure the luciferase activity using the luciferase assay system (Promega, Madison, WI, USA) as previously described [49]. Each experiment was performed in triplicate and repeated three times.

### WB and co-immunoprecipitation

WB was performed as previously described [50]. ACTIN was used as a loading control. The following primary antibodies were used: TC2N rabbit polyclonal antibody (1:500; Abcam), p53 mouse monoclonal antibody (1:1000; Santa Cruz Biotechnology), p21 mouse monoclonal antibody (1:1000; Santa Cruz Biotechnology), BAX mouse monoclonal antibody (1:1000; Santa Cruz Biotechnology), Bcl-2 mouse monoclonal antibody (1:1000; Santa Cruz Biotechnology), Cdk5 mouse monoclonal antibody (1:1000; Santa Cruz Biotechnology), phosphor-p53 (Ser-15) rabbit polyclonal antibody (1:500; Bioss), phosphor-p53 (Ser-33) rabbit polyclonal antibody (1:500; Bioss), phosphor-p53 (Ser-46) rabbit polyclonal antibody (1:500; Bioss), ubiquitin rabbit polyclonal antibody (1:500; Abcam) and ACTIN monoclonal antibody (1:2000; Sigma).

For co-immunoprecipitation, cell lysates were prepared and immunoprecipitated with indicated antibody or mouse IgG using the Co-IP Kit (Pierce, Rockford, IL, USA) according to the manufacturer’s instructions. The precipitated protein was assessed by WB using indicated antibody.

### Bioinformatics analysis

TCGA lung cancer RNAseq data (*n* = 1124) (https://cancergenome.nih.gov/) were used to compare the expression of TC2N with p53 and its target genes.

Kaplan–Meier curves were generated using the KMplot program (http://kmplot.com/analysis/) as previously described [2]. The patient samples were split into two groups to analyze the prognostic value of TC2N. Then, two patient cohorts were analyzed by Kaplan–Meier survival plot.

### Statistical analysis

Statistical analyses were performed with the SPSS 16.0 software (SPSS, Inc., Chicago, IL, USA). Each experiment was performed at least three times. Survival was analyzed by Kaplan–Meier and evaluated by log-rank test. Multivariate analysis of prognostic predictors was performed using Cox proportional hazard models. The data were presented as the means ± SD. Results of expression analyses, cell proliferation, cell apoptosis and cell cycle were analyzed using the two-tailed Student’s *t*-test. Correlation analysis of gene expression was performed using Spearman’s rank correlation coefficient analysis. A two-sided *P* value < 0.05 was taken as statistically significant.

## Electronic supplementary material

supplement information

Supplementary Figure S1

Supplementary Figure S2

Supplementary Figure S3

Supplementary Figure S4

Supplementary Figure S5

Supplementary Figure S6

Dataset 1
