# Supplementary material for: TC2N, a novel oncogene, accelerates tumor progression by suppressing p53 signaling pathway in lung cancer
Source: Cell Death Differ. 2018 Sep 25;26(7):1235–50. doi: 10.1038/s41418-018-0202-8 (PMC6748156; doi:10.1038/s41418-018-0202-8)
Supplement: Supplementary file 1 — supplement information [file 41418_2018_202_MOESM1_ESM.docx]

**Supplementary Figure legends**

**Supplementary Figure 1** TC2N is frequently overexpressed in lung cancer and is correlated with short survival time in lung cancer patients (**a**) TC2N mRNA expression in Selamat lung database. ***P<0.001. (**b**) TC2N mRNA expression in Okayama lung database. ***P<0.001. (**c**) Kaplan-Meier survival analysis of TC2N expression with OS in GSE3141 data set. (**d**) Kaplan-Meier survival analysis of TC2N expression with OS in GSE31210 data set. (**e**) Cox regression survival analysis of TC2N expression in 272 lung cancer patients split into two groups. (**f**) Cox regression survival analysis of TC2N expression in 272 lung cancer patients split into three groups. Patients with high TC2N protein expression were associated with a poor prognosis.

**Supplementary Figure 2** TC2N promotes proliferation and colony formation of lung cancer cells. (**a**) Photomicrographs of H460 and HBE cells at 46 h after TC2N inhibition or expression. EdU, 5-ethynyl-2’-deoxyuridine. Scale bars represent: 100μm (**b**) Colony formation assays were carried out in H460 cells expressing the negative control or shRNA of TC2N. The number of colonies or the number of cells was counted and compared in the diagrams. **P<0.01.

**Supplementary Figure 3** TC2N promotes proliferation and inhibits apoptosis of A549 and H1975 cell lines. (**a**) The overexpression of TC2N in A549 and H1975 cells were identified by WB assay. ACTIN serves as a loading control. MTS assays (**b**) and colony formation assays (**c**) were carried out in A549 and H1975 cells expressing the vector control or TC2N. The number of colonies or the number of cells was counted and compared in the diagrams. **P<0.01. (**d, e**) Flow cytometry assay were used to examine the effect of TC2N on cell cycle (**d**) and cell apoptosis (**e**). Error bars indicate s.d. (n = 3).

**Supplementary Figure 4** The expression of TC2N and EGFP were detected by WB in H460 stably cells. ACTIN serves as an internal control.

**Supplementary Figure 5** Ectopic expression of TC2N does not affected the Cdk5 mRNA expression. The Cdk5 mRNA expression was examined by qRT–PCR after knockdown of TC2N in H460 cells. ACTIN serves as an internal control.

**Supplementary Figure 6** The protein levels of P21 and BAX were monitored by WB after overexpression of TC2N in H1975 cells. ACTIN serves as an internal control.

**Supplementary Tables**

**Supplementary Table 1.** The correlation between TC2N expression and other signaling pathway-related genes.

| Pathway name | Gene name | R value | P value |
| --- | --- | --- | --- |
| Metabolism | PDK1 | -0.198 | <0.001 |
|  | LIPT2 | -0.110 | <0.001 |
|  | GCK | -0.220 | <0.001 |
|  | DUOX1 | -0.110 | <0.001 |
|  | GSTM2 | -0.281 | <0.001 |
|  | PKM2 | -0.250 | <0.001 |
|  | ALDOA | -0.113 | <0.001 |
|  | ENO1 | -0.105 | <0.001 |
|  | AMPD2 | -0.148 | <0.001 |
|  | PGK1 | -0.163 | <0.001 |
|  | GSTM4 | -0.255 | <0.001 |
|  | GSTA4 | -0.148 | <0.001 |
|  | ENO3 | 0.165 | <0.001 |
|  | ALDOB | 0.161 | <0.001 |
|  | CYP4F2 | -0.215 | <0.001 |
|  | CYP2W1 | -0.193 | <0.001 |
|  | CYP26B1 | -0.140 | <0.001 |
|  | CYP19A1 | -0.110 | <0.001 |
|  | LPA | -0.224 | <0.001 |
|  | … | … | … |
| Wnt | MYC | -0.286 | <0.001 |
|  | FOSL1 | -0.315 | <0.001 |
|  | CCND1 | 0.153 | <0.001 |
|  | TCF3 | -0.207 | <0.001 |
|  | LEF1 | -0.290 | <0.001 |
|  | FZD6 | -0.303 | <0.001 |
|  | WNT3A | -0.256 | <0.001 |
|  | … | … | … |
| VEGF | FGF2 | -0.173 | <0.001 |
|  | IL17RD | -0.201 | <0.001 |
|  | PSMF1 | -0.189 | <0.001 |
|  | MLST8 | -0.139 | <0.001 |
|  | DUSP16 | 0.205 | <0.001 |
|  | FGF16 | 0.100 | <0.001 |
|  | … | … | … |
| MAPK | MAPK1 | -0.164 | <0.001 |
|  | DUSP9 | -0.336 | <0.001 |
|  | PPP5C | -0.153 | <0.001 |
|  | PPP2R5D | -0.222 | <0.001 |
|  | MAP2K1 | -0.192 | <0.001 |
|  | MAPK3 | 0.146 | 0.014 |
|  | … | … | … |

**Supplementary Table 2.** Clinicopathologic characteristics of patients

|  | | |
| --- | --- | --- |
| Characteristic |  | Number of Patients |
| Patients |  | 272 |
| Sex |  |  |
| Male |  | 183 |
| Female |  | 89 |
| Age (years) |  | 30-86, median=63 |
| Tumor size (cm) |  | 1.0-9.0, median=3.0 |
| Histological type |  |  |
| Adenocarcinoma |  | 182 |
| Squamous cell carcinoma |  | 90 |
| Clinical stage (AJCC) |  |  |
| Ⅰ |  | 79 |
| Ⅱ |  | 75 |
| Ⅲ |  | 82 |
| Ⅳ |  | 3 |
| Histological grade |  |  |
| 1 |  | 22 |
| 2 |  | 187 |
| 3 |  | 63 |
| Lymph node metastasis |  |  |
| Yes |  | 124 |
| No |  | 121 |
| Clinical stage, tumor-nodes-metastasis, based on the American Joint Committee on Cancer/International Union Against Cancer Staging Manual (7th edition, 2009) | | |
|  |  |  |
|  |  |  |

**Supplementary Table 3**. The nucleotides applied in the study

| Description | Name | Sequence |
| --- | --- | --- |
| Primers for  qRT-PCR/RT-PCR  siRNA for P53  siRNA for Cdk5 | TC2N-F  TC2N-R  P53-F  P53-R  P21-F  P21-R  BAX-F  BAX-R  Bcl-2-F  Bcl-2-R  Cdk5-F  Cdk5-R  ACTIN-F  ACTIN-R  NC-siRNA  P53-siRNA  Cdk5-siRNA | TGGCTGTACTGAGGATTATTTGC  TGTGAAGGAGTTTCTTGTGTCC  CAGCACATGACGGAGGTTGT  TCATCCAAATACTCCACACGC  TGTCCGTCAGAACCCATGC  AAAGTCGAAGTTCCATCGCTC  CCCGAGAGGTCTTTTTCCGAG  CCAGCCCATGATGGTTCTGAT  GGTGGGGTCATGTGTGTGG  CGGTTCAGGTACTCAGTCATCC  GGAAGGCACCTACGGAACTG  GGCACACCCTCATCATCGT  CCACGAAACTACCTTCAACTCC  GTGATCTCCTTCTGCATCCTGT  5'-UUCUCCGAACGUGUCACGUUU-'3  5'-GACUCCAGUGGUAAUCUACTT-'3  5'-CCTGCTAATAAACAGGAAT-'3 |
