# Supplementary figures and images for: TC2N, a novel oncogene, accelerates tumor progression by suppressing p53 signaling pathway in lung cancer
Source: Cell Death Differ. 2018 Sep 25;26(7):1235–50. doi: 10.1038/s41418-018-0202-8 (PMC6748156; doi:10.1038/s41418-018-0202-8)

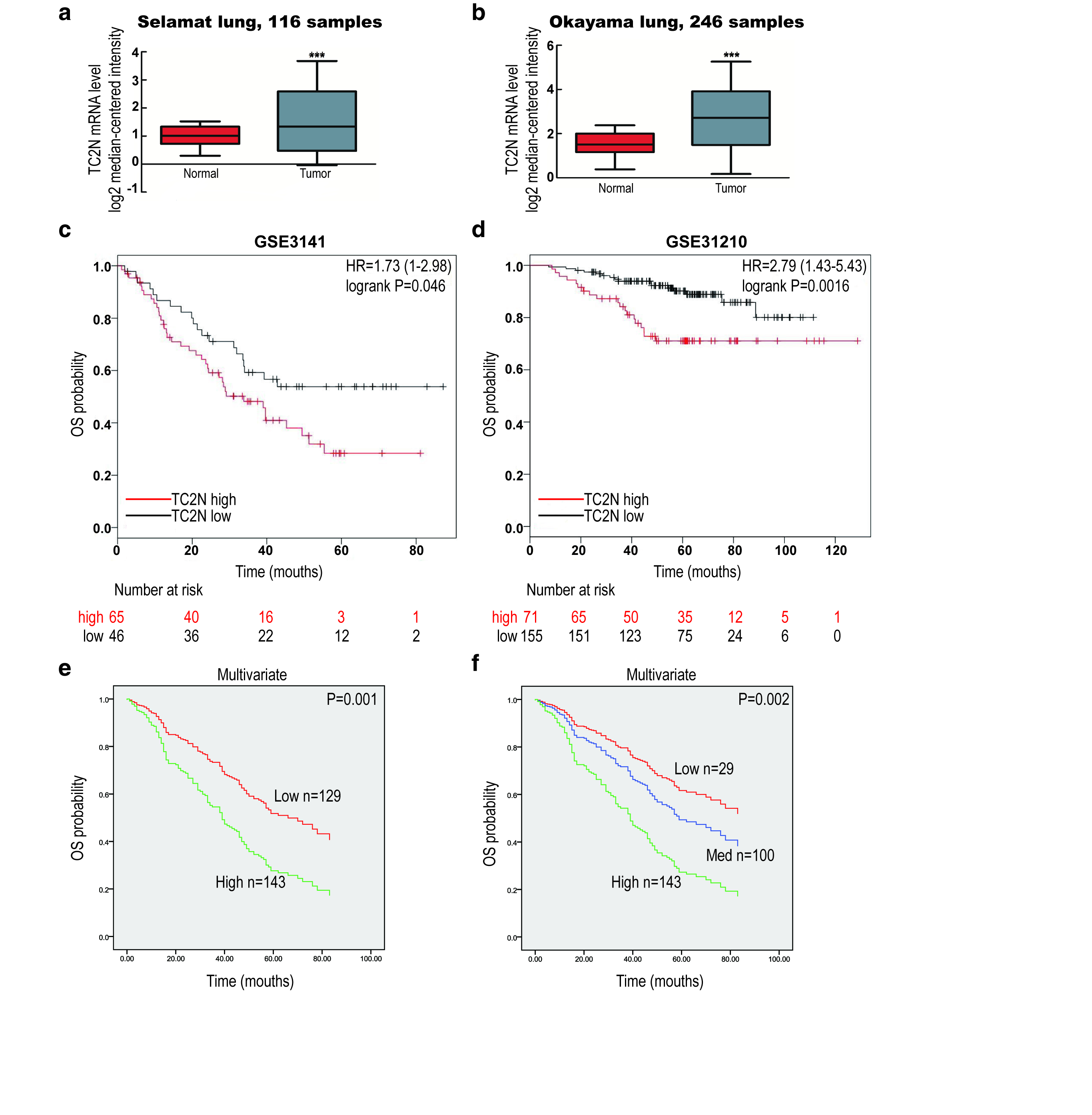

Supplement: Supplementary file 2 — Supplementary Figure S1 [file 41418_2018_202_MOESM2_ESM.tif]

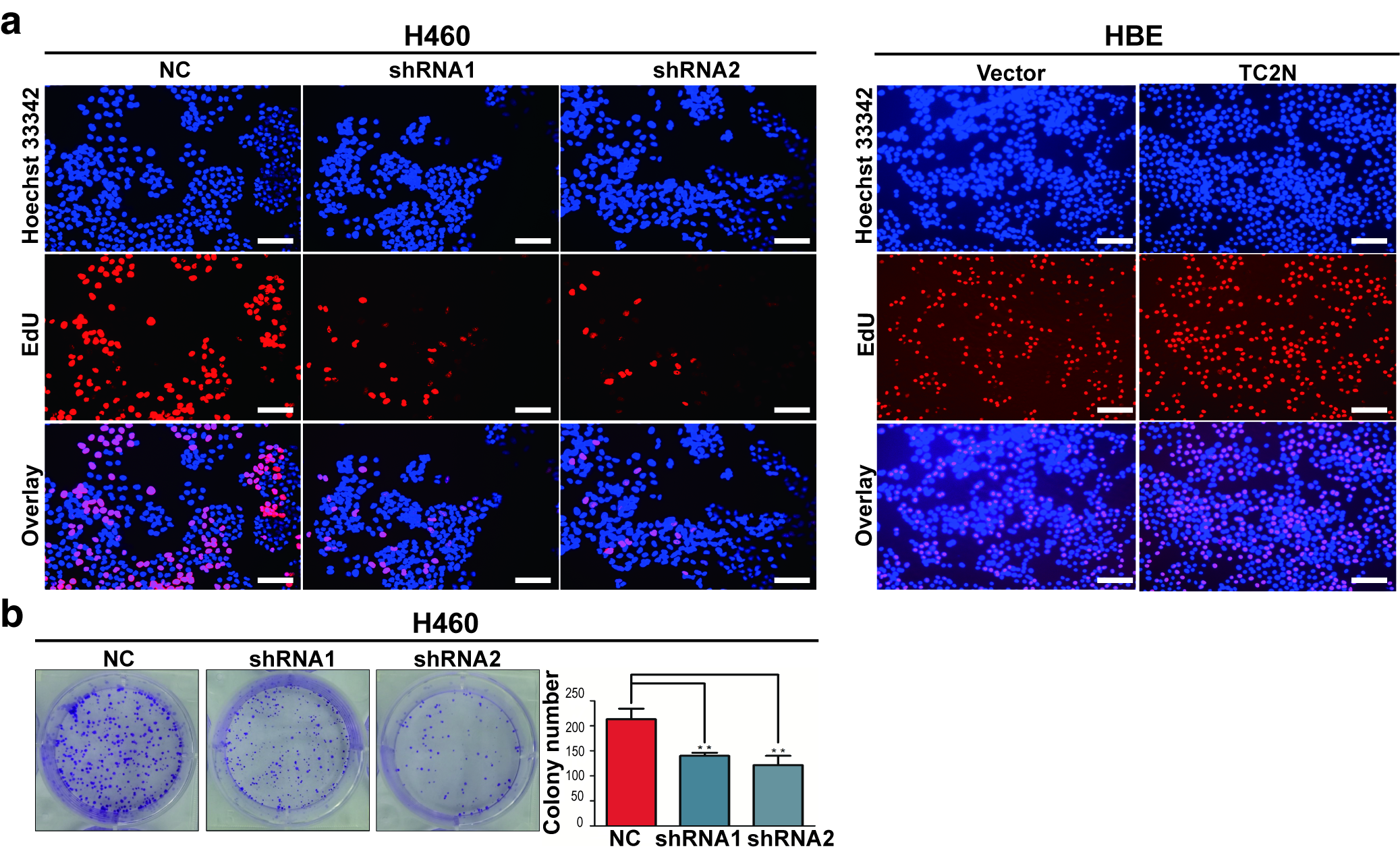

Supplement: Supplementary file 3 — Supplementary Figure S2 [file 41418_2018_202_MOESM3_ESM.tif]

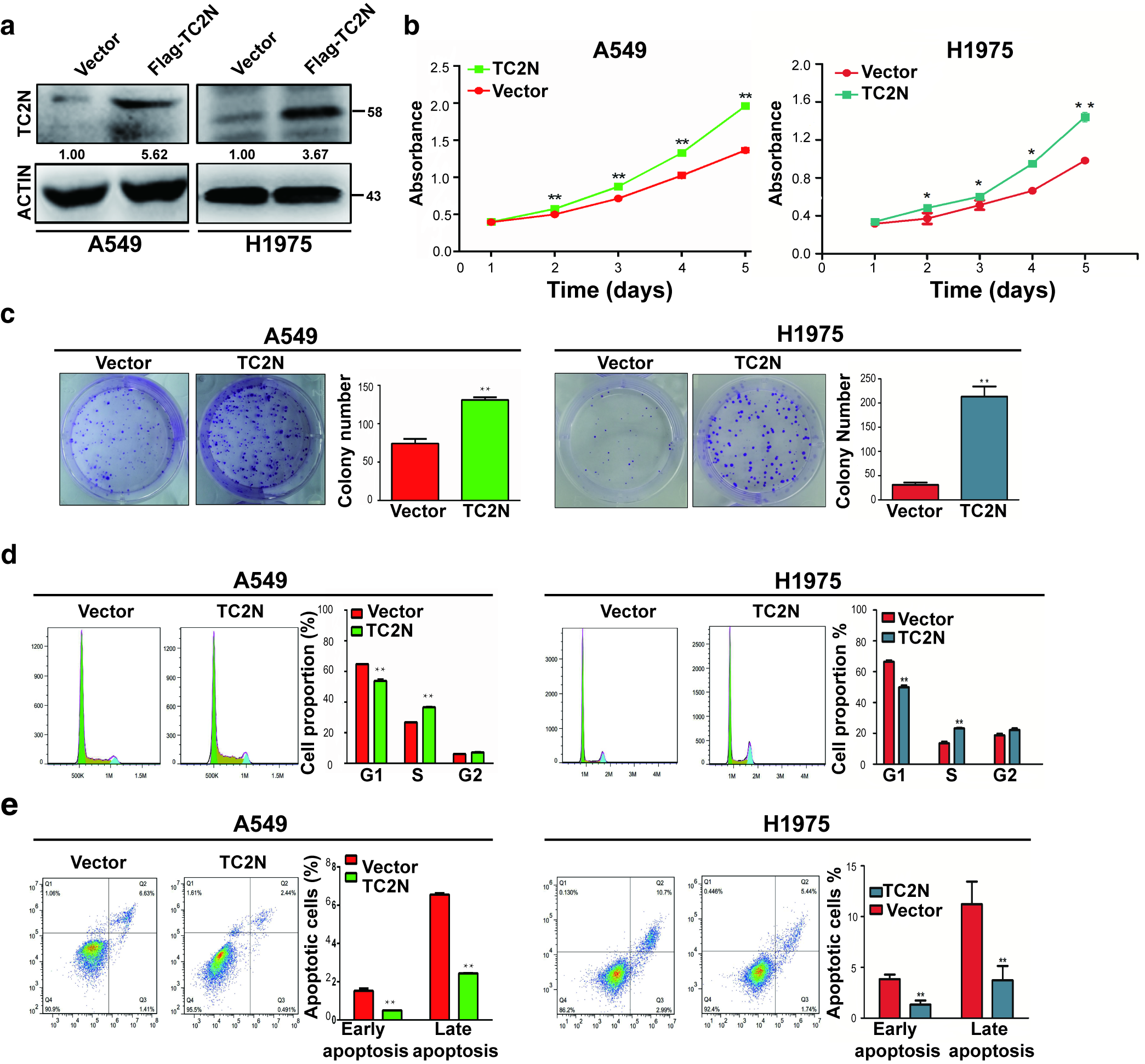

Supplement: Supplementary file 4 — Supplementary Figure S3 [file 41418_2018_202_MOESM4_ESM.tif]

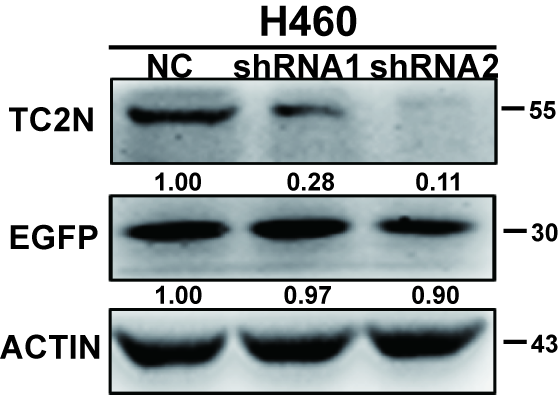

Supplement: Supplementary file 5 — Supplementary Figure S4 [file 41418_2018_202_MOESM5_ESM.tif]

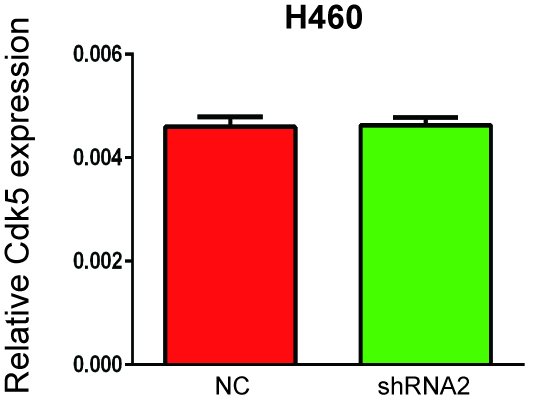

Supplement: Supplementary file 6 — Supplementary Figure S5 [file 41418_2018_202_MOESM6_ESM.tif]

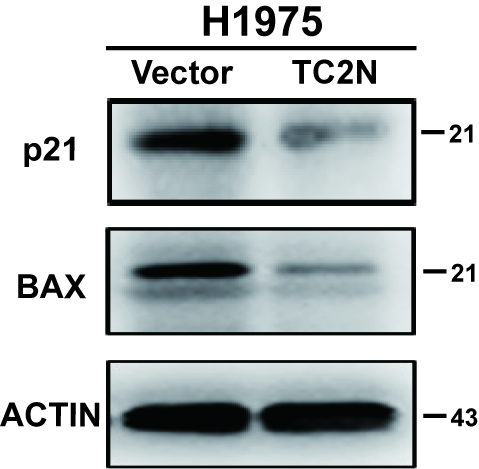

Supplement: Supplementary file 7 — Supplementary Figure S6 [file 41418_2018_202_MOESM7_ESM.tif]
